# Adjuvant Bidirectional Chemotherapy Using an Intraperitoneal Port

**DOI:** 10.1155/2012/752643

**Published:** 2012-07-24

**Authors:** Paul H. Sugarbaker, Lana Bijelic

**Affiliations:** Program in Peritoneal Surface Malignancy, Washington Cancer Institute, Washington, DC 20010, USA

## Abstract

Cytoreductive surgery (CRS) and hyperthermic intraperitoneal chemotherapy (HIPEC) have been established as treatment options for patients with peritoneal metastases or peritoneal mesothelioma. However, this novel treatment strategy remains associated with a large percentage of local-regional treatment failures. These treatment failures are attributed to the inadequacy of HIPEC to maintain a surgical complete response. Management strategies to supplement CRS and HIPEC are indicated. A simplified approach to the intraoperative placement of an intraperitoneal port for adjuvant bidirectional chemotherapy (ABC) was devised. Four different chemotherapy treatment plans were utilized depending upon the primary site of the malignancy. Thirty-one consecutive patients with an intraoperative placement of the intraperitoneal port were available for study. The incidence of adverse events that caused an early discontinuation of the bidirectional chemotherapy occurred in 75% of the 8 patients who had an incomplete cytoreduction and in 0% of patients who had a complete cytoreduction. All of the patients who had complete cytoreduction completed at least 5 of the scheduled 6 bidirectional chemotherapy treatments. Adjuvant bidirectional chemotherapy is possible following a major cytoreductive surgical procedure using a simplified method of intraoperative intraperitoneal port placement.

## 1. Introduction

Cancer chemotherapy can be given through a number of different routes of administration. Although intravenous delivery is most common, intraperitoneal, intrapleural, and intrathecal chemotherapy infusions have been utilized with good results. Also, intra-arterial perfusion of chemotherapy has been reported as successful by several groups. The low incidence of complications with simple intravenous drug delivery most likely accounts for its more common utilization. However, in some specific situations, intraperitoneal drug delivery, or intraperitoneal drug delivery combined with intravenous drug delivery have been definitely shown to improve outcome. In patients with ovarian cancer, three prospective and randomized studies with combined intraperitoneal and intravenous chemotherapy compared to only intravenous chemotherapy have consistently shown an improvement in long-term survival with the local-regional approach [1–3]. In patients with ovarian cancer that was resected so that all tumor masses greater than 2 cm were removed, survival was significantly longer in 546 randomized patients in those who received intraperitoneal cisplatin as compared to intravenous cisplatin (*P* = 0.02). Also, moderate to severe nervous system toxicity was reduced with the intraperitoneal cisplatin [1].

Many oncologists acknowledge that disease control may be significantly improved when chemotherapy is administered through the intraperitoneal route [4]. However, they are aware that the complications of intraperitoneal chemotherapy administration are frequent and occasionally life endangering [5]. There are, of course, adverse events with the use of intravenous ports that are used in a large proportion of patients receiving systemic cancer chemotherapy. Nevertheless they are used as standard of care. In contrast, the difficulties that may occur with placement of an intraperitoneal port, the patient discomfort that frequently accompanies chemotherapy administration, and the serious life endangering complications sometimes requiring reoperation discourage its routine use [6].

Successful randomized trials testing combinations of intraperitoneal and intravenous chemotherapy for gastrointestinal peritoneal metastases and for peritoneal mesothelioma have not been performed to date. However, the rationale for such an approach is strong. In this paper, we describe a new and simplified method for placement of an intraperitoneal port following cytoreductive surgery and heated intraoperative intraperitoneal chemotherapy. Our clinical experience with 31 consecutive patients having adjuvant bidirectional chemotherapy for peritoneal mesothelioma or gastrointestinal carcinomatosis from a variety of primary sites is reported.

## 2. Materials and Methods

All patients in this retrospective paper had peritoneal metastases documented within the abdomen and pelvis. They underwent cytoreductive surgery with an attempt to clear all of the malignancy from the abdomen. Following this, they were treated with a perioperative chemotherapy treatment using heated intraperitoneal or a combination of heated intraperitoneal and intravenous chemotherapy.

### 2.1. Preparation for Intraperitoneal Port Placement

Following completion of the cytoreductive surgery and hyperthermic intraperitoneal chemotherapy, the abdomen and pelvis were again widely exposed. All intestinal reconstruction was completed. The abdomen was irrigated with 4 liters of a warm saline (37°C) solution. The irrigation solution contained the antibiotics neomycin and polymyxin B (XGen Pharmaceuticals, Big Flats, NY). The abdominal skin was again cleansed with a povidone iodine solution.

### 2.2. Technique for Intraperitoneal Port Placement

An 8 cm incision was made at the lateral aspect of the left rectus muscle. This transverse incision was in line with the lowest aspect of the ribcage. It was continued through the subcutaneous tissue to the anterior rectus sheath. At the lateral aspect of the rectus muscle, the external oblique aponeurosis was incised.

**Figure 1:**
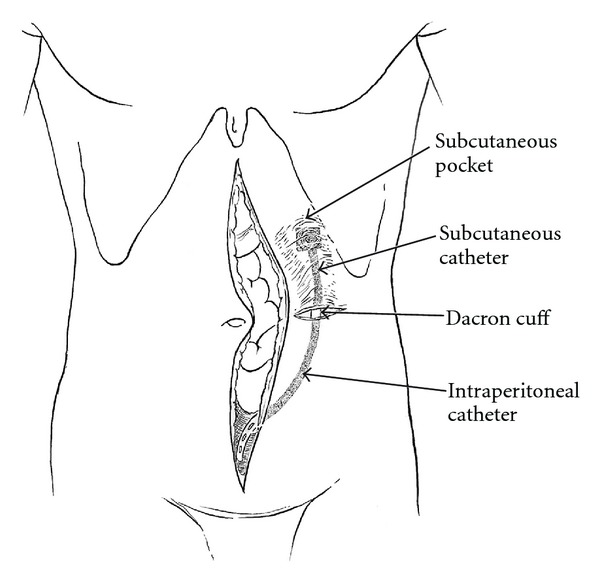
A lateral skin incision allows dissection of the port pocket and access to the abdomen using a stab incision.

From this incision, subcutaneous tunnel and pocket for an intraperitoneal port system were constructed (Port-A-Cath, Smiths Medical MD, Inc., St. Paul, MN, USA). The port was located superior to and directly over the superior portion of the left rectus muscle. Care should be taken not to enter the abdominal incision with the tunnel or port pocket (Figure 1).

Through the incision in the external oblique fascia, a tonsil clamp is positioned, moving from the peritoneal cavity to the subcutaneous space with the stab incision. The clamp guides the catheter tip into the midabdomen. The tip is directed toward the jejunal loops of the small bowel. The Dacron cuff is secured with a resorbable purse string suture to the external oblique aponeurosis.

**Figure 2:**
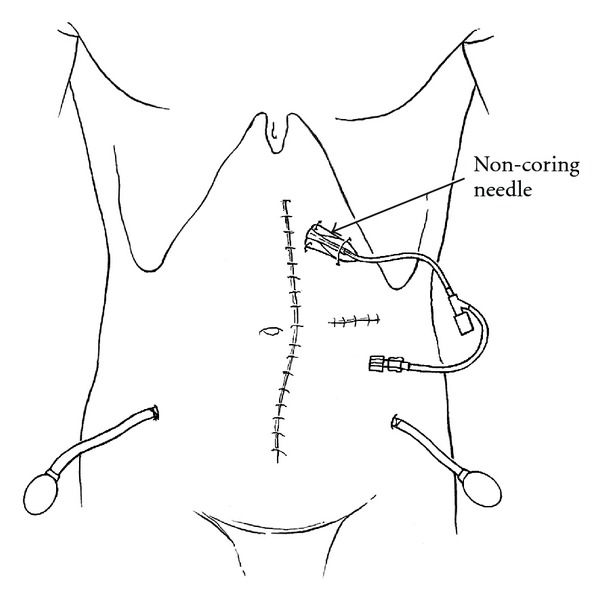
A noncoring needle is used to maintain optimal position of the port for 10 days.

The catheter is cut to an appropriate length and secured to the port. The port is advanced through the tunnel into its pocket. The port is secured manually in its proper position and accessed with a noncoring right angle needle (Port-A-Cath, Gripper Plus, Deltec, Inc., St. Paul, MN, USA). The port and tubing are flushed with saline solution by irrigating the noncoring needle. Following this, the needle is capped off with a male adapter. The plastic base of the right angle non-coring needle is secured at its four corners with a 2–0 nylon suture (Figure 2).

The tunnel and incision are copiously irrigated with the antibiotic solution and hemostasis checked. Scarpa's fascia is closed over the Dacron cuff with a resorbable suture and the skin closed with interrupted nonabsorbable sutures. The noncoring needle is covered by an occlusive gauze dressing.

Liberal placement of Seprafilm (Genzyme Biosurgery, Framingham, MA) on abdominal and pelvic surfaces devoid of parietal peritoneum and between the loops of small bowel is recommended.

At this point, the abdominal incision is closed. The port access with the Huber needle is retained for 10 days to ensure proper position of the port for easy access for the adjuvant bidirectional chemotherapy (ABC) treatments.

### 2.3. Chemotherapy Regimens Utilized

Four different combined intraperitoneal and intravenous chemotherapy regimens were utilized for different diseases treated by the ABC method. For peritoneal mesothelioma, a combination of intraperitoneal pemetrexed with intravenous cisplatin was used. For appendiceal or colorectal malignancy, a combination of intraperitoneal 5-fluorouracil and systemic oxaliplatin was used. For ovarian cancer, a combination of intraperitoneal paclitaxel and systemic cisplatin was used. Finally, for the pancreas cancer patients, intraperitoneal gemcitabine was used. No intravenous chemotherapy was combined with the intraperitoneal gemcitabine (see Table 1).

The selection criteria for intraoperative placement of the intraperitoneal port was variable depending on the patient's diagnosis. All pancreas cancer patients during the study period had an intraperitoneal port placed if the R0 pancreaticoduodenectomy operation could be completed. All peritoneal mesothelioma patients had port placement if a complete or near complete cytoreduction was possible. The same was true with the three papillary serous malignancy patients. In patients with appendiceal adenocarcinoma, intraoperative port placement was utilized if systemic treatment options had been exhausted. The same was true with rectal cancer patients.

## 3. Results

There were 31 patients treated using an intraperitoneal port placed following the completion of cytoreductive surgery and hyperthermic intraperitoneal chemotherapy. Five patients had a diagnosis of appendiceal adenocarcinoma, 19 had peritoneal mesothelioma, 3 had pancreas cancer, 1 had rectal cancer, and 3 had papillary serous cancer. The median age on these patients was 49 with a range from 32 to 74. Twenty-three patients had complete or near complete (adequate) cytoreductive surgery prior to port placement. Eight patients had an incomplete cytoreduction.

Six major events occurred in eight patients (75%) who had incomplete cytoreduction. Four patients had disease progression, 1 patient had bowel perforation, and 1 patient had a port occlusion after 3 cycles which was not remedied and intraperitoneal treatments ceased. In these six patients, the adverse event resulted in a discontinuation of the ABC.

In the 23 patients who had complete or near complete cytoreduction, there were 6 events. One patient had systemic progression at cycle 3 and the combined intraperitoneal and intravenous chemotherapy, was discontinued. One patient developed methicillin-resistant Staphylococcus aureus infection of the port postoperatively. In these two patients (9%) the adverse event resulted in discontinuation of the ABC.

Four patients had events which did not significantly impede or disrupt their chemotherapy treatments, 1 patient had port occlusion after 5 cycles so that the final cycle of ABC was given systemically. One patient had a port occlusion successfully treated by laparoscopic intervention and successfully completed the ABC. One patient had an infected port which was removed; one cycle of pemetrexed and cisplatin chemotherapy was given intravenously and then the port replaced and the bidirectional treatment completed. One patient required hospitalization after ABC treatments on 3 occasions. His final cycle of pemetrexed and cisplatin was then given systemically. One patient had port infection when on second line intraperitoneal chemotherapy and her adverse event (peritonitis) was not included in these statistics.

## 4. Discussion

### 4.1. Developmental Plan for Adjuvant Bidirectional Chemotherapy

The ABC regimens used on these patients were designed from pharmacologic data obtained in chemotherapy agents known to show a response in the primary disease to be treated. Also, morbidity and mortality testing showed that the doses and schedules of drugs used were safe [7]. The effectiveness of these combined intraperitoneal and intravenous treatments has not been tested in a randomized study against their intravenous counterparts. This second important step in the development of the ABC approach has yet to be initiated.

### 4.2. Need for Complete Cytoreduction

By these early data, patient selection for ABC treatment is shown to be necessary. The clinical correlate most impressive was the impact of completeness of cytoreduction on the likelihood of completing the prescribed chemotherapy. Seventy-five percent of those patients who had gross disease after cytoreduction (CC-3) did not complete their scheduled treatments. The most common reason for this was disease progression. A majority of patients with complete cytoreduction received at least 5 of their 6 treatments using bidirectional administration.

### 4.3. Advantages of Intraoperative versus Delayed Intraperitoneal Port Placement

There may be advantages of intraoperative placement of the peritoneal port. A major advantage concerns placement of the port directly between jejunal bowel loops. This precise anatomic placement is difficult and usually impossible with a postoperative port placement. Secondly, it is much more acceptable to patients in that it does not require another operative intervention. Also, there may be bowel perforation with a delayed placement of the intraperitoneal port. This serious problem is avoided.

### 4.4. Precautions to Prevent Infection

A possible disadvantage of intraoperative placement of an intraperitoneal port reported in the literature is a higher infection rate. We have combated this by large volume irrigation of the abdominal space with an antibiotic solution following cytoreductive surgery. Also, not only is the abdomen cleansed with a large volume of irrigation but the skin is reprepared with povidone iodine before the catheter is brought onto the operative field.

### 4.5. Prolonged Port Stabilization with a Non-Coring Needle after Placement in Its Pocket

Needle access to the port is maintained over ten days in order to allow fixation of the port within its tunnel. No non-absorbable sutures are used at the four corners of the port in order to stabilize it within the pocket. This eliminates the need for an incision close to the port for placement of nonabsorbable sutures at each corner. The port stabilization by a non-coring needle has not caused us any problems with infection. Of course, this also facilitates removal of the port at a later time.

### 4.6. Position of Port in Left Subcostal Space

Our technique is considerably different in terms of the anatomic placement of the port than other techniques. The port is placed in the subcostal space in the upper left portion of the abdomen. The base of the port is stabilized by the anterior rectus sheath and flexion of the rectus muscle by the patients gives a solid base for access with the non-coring needle. Making a long tunnel up to the ribcage is unnecessary. Also, this long tunnel and port placement on the chest wall is uncomfortable for patients.

### 4.7. Factors That May Impact on Satisfactory Port Function

In these data, the only clinical feature that had an impact on satisfactory port function was the completeness of cytoreduction. Undoubtedly, there are other factors which, in a larger study, will be shown to influence the long-term function of the port. It is possible that more port or catheter infections will occur in those patients who have had a bowel anastomosis or some other potential contamination of the peritoneal space by enteric organisms. It is possible that the extent of peritonectomy, and therefore the extent of intra-abdominal adhesions, will be important in long-term function. In the patients in this study, all had very extensive cytoreduction and therefore data regarding the extent of cytoreduction was not available. It is possible that the use of adhesion-prevention agents may be important. For example, the liberal use of Seprafilm to cover peritonectomy sites may be advisable. Also, Seprafilm can be used between the loops of small bowel and its mesentery. Alternatively, the use of early postoperative intraperitoneal 5-fluorouracil or paclitaxel may reduce the extent of abdominal and pelvic adhesions and thereby facilitate more adequate long-term port function [8]. Finally, in this study we only gathered data on those patients who had an intraoperative placement of the intraperitoneal port. Whether this placement is best performed in the operating room with the cytoreductive intervention or later on following full recovery from surgery has yet to be determined.

### 4.8. A Unique Phase II Study

In a survey of the literature regarding the use of an intraperitoneal port, no prior data regarding port placement after CRS and HIPEC was found. This is the first phase II study that attempts to prospectively gather clinical information on port insertion along with the definitive cytoreductive intervention. ABC is feasible using this methodology, and it was thought to be acceptable to patients with a small inconvenience. Trials to test ABC versus traditional systemic chemotherapy may now be appropriate.

### 4.9. Advantages of Combined Intraperitoneal and Intravenous (Bidirectional) Treatments

Theoretically, it would be possible to administer all of the chemotherapy agents presented in Table 2 by the intraperitoneal route as opposed a bidirectional treatment as proposed in this review. We did not mix drugs for simultaneous two-drug infusions for several reasons. First of all, there are issues with drug incompatibility. For example, 5-fluorouracil cannot be mixed with other drugs because of problems with precipitation. Also, the safety of two drugs simultaneously administered into the peritoneal cavity has not been previously explored. Phase I protocols to test the safety of two drugs administered simultaneously into the peritoneal cavity would be necessary. Perhaps most importantly, pharmacologic data suggests that drugs administered intravenously with an artificial ascites will target the peritoneal surfaces [9]. Van der Speeten and colleagues showed that patients who received intravenous 5-FU along with a volume of intraperitoneal fluid maintained a higher level of 5-FU in the peritoneal space as compared to the intravenous drug levels over a prolonged time period. The area under the curve ratio of peritoneal fluid to plasma was 2.3. These data suggest that intravenous drugs can be targeted to the peritoneal surface if administered simultaneously with a large volume of intraperitoneal chemotherapy solution.

## Figures and Tables

**Table 1:** Clinical data on 31 consecutive patients given chemotherapy through a permanent intraperitoneal port placed prior to the closure of the abdomen.

Gender	
Male	16
Female	15
Age	
Median	49
Range	32–74
Diagnosis	
Peritoneal mesothelioma	19
Appendiceal adenocarcinoma	5
Papillary serous cancer	3
Pancreas cancer	3
Rectal cancer	1
Cytoreduction	
Complete or near complete (CC-0/CC-1)	23
Incomplete cytoreduction	8
% of patients completing 5 or more cycles of with adverse events requiring removal of intraperitoneal port	
Complete or near complete cytoreduction	9% (2/23)
Incomplete cytoreduction	75% (6/8)

**Table 2:** Four different combined intraperitoneal and intravenous chemotherapy (bidirectional) treatment options.

Disease	Combined intraperitoneal and intravenous chemotherapy treatment option
Peritoneal mesothelioma	Pemetrexed (500 mg/m^2^) in 1000 mL 1.5% dextrose peritoneal dialysis solution as a 60-minute rapid infusion through the intraperitoneal port. Cisplatin (75 mg/m^2^) in 250 mg of normal saline is given over 120 minutes immediately following the pemetrexed infusion.

Adenocarcinoma	5-fluorouracil (600 mg/m^2^) in 1000 mL 1.5% dextrose peritoneal dialysis solution through the intraperitoneal port with the administration as rapid as possible. After the intraperitoneal chemotherapy infusion is complete, oxaliplatin (130 mg/m^2^) in 250 mL of dextrose in water is given as a 2-hour intravenous infusion.

Pancreas cancer	Gemcitabine (1000 mg/m^2^) in 1000 mL 1.5% dextrose peritoneal dialysis solution through the intraperitoneal port as rapid as possible is given on days 1, 8, and 15 of a 4-week cycle.

Papillary serous and ovarian cancer	Paclitaxel (20 mg/m^2^) in 1000 mL 6% Hetastarch through the intraperitoneal port. Intravenous cisplatin (75 mg/m^2^) is given after the paclitaxel infusion is complete over 120 minutes.
